# Bipartite Graphs as Models of Population Structures in Evolutionary Multiplayer Games

**DOI:** 10.1371/journal.pone.0044514

**Published:** 2012-09-10

**Authors:** Jorge Peña, Yannick Rochat

**Affiliations:** Institute of Applied Mathematics, Faculty of Social and Political Sciences, University of Lausanne, Lausanne, Switzerland; Universidad Carlos III de Madrid, Spain

## Abstract

By combining evolutionary game theory and graph theory, “games on graphs” study the evolutionary dynamics of frequency-dependent selection in population structures modeled as geographical or social networks. Networks are usually represented by means of unipartite graphs, and social interactions by two-person games such as the famous prisoner’s dilemma. Unipartite graphs have also been used for modeling interactions going beyond pairwise interactions. In this paper, we argue that bipartite graphs are a better alternative to unipartite graphs for describing population structures in evolutionary multiplayer games. To illustrate this point, we make use of bipartite graphs to investigate, by means of computer simulations, the evolution of cooperation under the conventional and the distributed N-person prisoner’s dilemma. We show that several implicit assumptions arising from the standard approach based on unipartite graphs (such as the definition of replacement neighborhoods, the intertwining of individual and group diversity, and the large overlap of interaction neighborhoods) can have a large impact on the resulting evolutionary dynamics. Our work provides a clear example of the importance of construction procedures in games on graphs, of the suitability of bigraphs and hypergraphs for computational modeling, and of the importance of concepts from social network analysis such as centrality, centralization and bipartite clustering for the understanding of dynamical processes occurring on networked population structures.

## Introduction

Since the pioneering work of Maynard Smith and Price [1], evolutionary game theory [2] has become a valuable tool to describe and study evolutionary dynamics when fitness is frequency-dependent. Evolutionary game theory builds on the theory of games [3] by considering populations of individuals whose success or fitness depends on the outcome of social interactions. Behavioral strategies are genetically or culturally inherited, so that the relative abundance of fitter strategies increases over time due to natural selection or social learning. When populations are assumed to be infinite and well-mixed, the replicator dynamics [4], [5] offers a deterministic and exact account of the evolutionary dynamics.

In spite of the importance of the replicator dynamics as a mathematical tool for investigating evolutionary dynamics, it is obvious that real populations are never infinite nor perfectly well-mixed. Games on graphs (see Refs. [6] and [7] for reviews) go beyond these two simplifying assumptions by considering finite-sized populations embedded in graphs representing geographical isolation or social networks. A graph 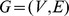 consists of a set 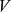 of vertices and a set 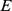 of edges connecting pairs of vertices. In general models of games on graphs, individuals are placed on two graphs with the same set of vertices [8]: the interaction graph 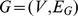 and the replacement graph 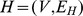. Evolutionary dynamics are specified so that, first, individuals play two-person games with their neighbors in the interaction graph 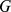, and second, strategy updating takes place along the edges of the replacement graph 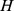. Although the set of edges of the replacement graph may differ from the set of edges of the interaction graph, it is usually assumed that 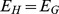 so that 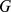 and 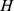 effectively coincide.

A perusal of the vast literature on games on graphs highlights the importance of network structure in the evolutionary dynamics of different games. Two particular results are worth mentioning. First, although unconditional cooperation under the one-shot prisoner’s dilemma (PD) is not evolutionarily stable in infinite and well-mixed populations, it can be viable in sparse homogeneous networks [9]–[11]. “Spatial reciprocity” [12], “network reciprocity” [13] and “graph selection” [14] are different labels that have been coined in order to contrast such effect with other cooperation-promoting mechanisms (see, however, Refs. [11], [15]–[17] for the close connections between network reciprocity and kin selection via limited dispersal, and between games on graphs and inclusive fitness theory). Second, heterogeneous population structures such as scale-free networks [18] can significantly promote cooperation under the PD and other social dilemmas [19], [20], although such promotion strongly depends on several details of the network, the payoff functions and the updating rules [7], [21]–[26].

Notwithstanding the importance of pairwise social interactions, many situations in real social systems require the collective action of groups comprised by more than two individuals. Moreover, interactions within these larger groups can not always be represented as disjointed collections of two-person games [27]. Public goods games (PGGs) are paradigmatic among such non-decomposable multiplayer games. PGGs are models of situations where individuals face the dilemma of providing and/or maintaining a public good: a common resource that is both non-excludable (no individual can be excluded from its consumption) and non-rivalrous (one individual’s use of the public good does not diminish its availability to another individual) [28]. Digestive enzymes in yeast [29], ATP in heterotrophic microorganisms [30], webs in social spiders [31], alarm calls in meerkats [32], collective hunting in lions [33], and open-source software in contemporary humans [34] are typical examples of public goods whose abusive exploitation by non-contributing individuals may lead to the so-called tragedy of the commons [35]: a situation in which nobody contributes and therefore no public good is produced or maintained.

By far, the most well known PGG is the N-person prisoner’s dilemma (NPD) [36]. In this game, each individual in a group of size 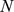 has to decide whether to cooperate (by contributing to a common pot) or to defect (by refraining from contributing). The sum of the individual contributions is multiplied by a factor 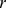 and then equally distributed among all players, including those who did not contribute. No matter the decisions taken by the other players, it is always better to defect if 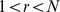. In infinite well-mixed populations and in the absence of cooperation-promoting mechanisms, defection is evolutionarily stable and the replicator dynamics predicts the ultimate extinction of Cs. However, as it is also the case in the two-person PD, cooperation in the NPD can be sustained in structured populations under particular life-cycle assumptions. Hauert et al. [37] studied a spatial NPD resulting from placing the individuals in the nodes of a two-dimensional lattice and restricting interactions to nearest neighbors. In this model and for large values of 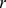 (but still for 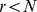) Cs are able to survive by minimizing interactions with Ds through cluster formation. Hauert et al.’s model has been extended by Santos et al. [38], who used scale-free networks instead of regular lattices as population structures. The highly heterogeneous degree distributions of scale-free networks introduce *social diversity* both at the individual level (players vary greatly with respect to the number of games they take part in) and at the group level (different games are played by different numbers of players). Social diversity brings up a moderate promotion of cooperation when Cs pay a fixed cost 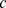 per game, but a significant boost when Cs pay a fixed cost 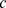 for all the games they play. In the following we use the terminology introduced in Ref. [39] and call conventional NPD the former case and distributed NPD the latter.

The way networks are constructed is a common feature of Refs. [37], [38] and many other papers dealing with evolutionary multiplayer games on networks [40]–[52]. We refer to this construction procedure as *the graph approach*. According to this framework, nodes of a graph 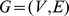 define both individuals playing a game and games being played by the focal individual plus its direct neighbors, so that an individual with 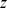 neighbors takes part in 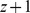 games: the one centered on itself plus 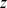 games, each centered on one of its neighbors. Fitness or social success is given by the sum of payoffs collected in these 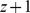 games, and competition or imitation takes place along the edges of the graph.

**Figure 1:**
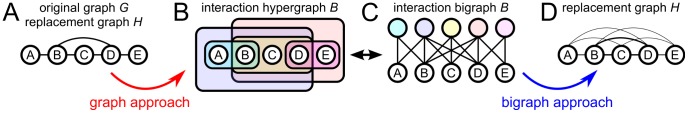
Modeling population structures in evolutionary multiplayer games. The graph approach consists in first defining the original graph 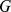 (Panel A) and then constructing the interaction hypergraph 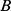 (Panel B) by associating a hyperedge with each closed neighborhood in 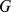. The interaction hypergraph can be also represented as a bigraph (Panel C), where individuals/players are bottom vertices and groups/games are top vertices. In the graph approach the replacement graph 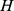 is assumed to be equal to the original graph 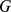, so that interactions take place along the hyperedges of the hypergraph, but strategy updating occurs along the edges of the original graph. The alternative bigraph approach consists in first defining the interaction bigraph 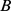 (Panel C) and then obtaining the replacement graph 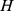 (Panel D) as the bottom projection of the interaction bigraph. Weights can be attached to the links of the replacement graph according to different heuristics (here, the “unnormalized weighted projection” method is used; the width of the links is proportional to the links’ weights). The interaction bigraph can be constructed from a bipartite graph model or following the graph approach from a simple graph 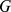 (Panel A). In this last case, the replacement graphs due to the graph approach (the original graph shown in Panel A) and to the bigraph approach (the projection of the interaction bigraph shown in Panel D) differ, the latter being denser.

An alternative way of looking at the population structure resulting from the graph approach is realizing that while the replacement graph is the *original graph* 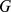, the interaction graph is actually a hypergraph (or a bipartite graph) in which hyperedges (or top vertices) correspond to closed neighborhoods of 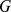 (see Figure 1). A hypergraph is the generalization of a graph for the case where edges (called in this case hyperedges) can connect arbitrarily many vertices. A bipartite graph 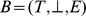, also called a bigraph, consists of two disjoint sets of vertices, 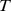 (top vertices) and 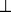 (bottom vertices), and a set of edges, 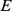. The difference between bipartite graphs and standard unipartite graphs is that edges in a bigraph only connect vertices of different kinds. Undirected hypergraphs and bigraphs are mathematically equivalent, but bigraphs are usually easier to implement and to work with. Many real biological and social networks display a natural bipartite structure and can be represented as bigraphs in a straightforward manner. Food webs [53] and metabolic networks [54] are well known biological examples; social examples include affiliation [55] or collaboration networks [56], such as those connecting co-owners of companies [57], film actors [58] and scientists [56]. In this paper, we represent groups/games as top vertices and individuals/players as bottom vertices. Three network statistics will be particularly important. First, the *top degree distribution* gives the distribution of the number of games being played by a given individual. Second, the *bottom degree distribution* gives the distribution of the number of individuals playing a given game. Finally, the *bipartite clustering coefficient* captures correlations between the neighborhoods of bottom vertices, i.e. the degree to which groups overlap.

With the previous definitions, the graph approach can be interpreted as one in which (1) the replacement graph is defined, and (2) the interaction bigraph is constructed from the replacement graph. This approach has become the de facto standard for modeling population structures in multiplayer games on graphs. However, it has many important limitations. First, since both players and games are identified with the same set of vertices, the numbers of games and players are exactly the same, i.e. 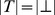 in the resulting interaction bigraph. Second, and for the same reason, the top degree distribution and the bottom degree distribution coincide. In real systems, however, these distributions are usually very different. In collaboration networks, for example, the number of papers per author has been shown to follow a power-law distribution while the number of authors per paper generally follows an exponential distribution [56]. Third, the graph approach automatically leads to a relatively large bipartite clustering coefficient. Although such large coefficient seems to be an intrinsic property of many social and biological networks [59], its presence by default in models of games on graphs can be a drawback if the goal is to build null models of connectivity patterns or to study the effects of bipartite clustering. Fourth, while each individual effectively interacts with second-order neighbors in the original graph, strategy updating is posited to occur only between first-order neighbors. As an example of this, consider the graphs depicted in Panels A and B of Figure 1. Note that individual 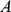 plays with 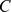 and 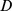 the game centered on 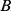, but 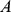 is not connected to 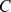 nor 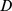 in the replacement graph. Finally, replacement graphs in the graph approach do not reflect encounter rates between two individuals but rather assume that all neighbors in the replacement graph are equally important. Consider again the graphs depicted in Panels A and B of Figure 1. On the one hand, individual 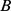 plays thrice with 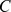 (the games centered on 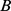, 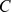 and 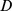) but only twice with 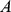 (the games centered on 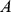 and 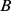). On the other hand, individual 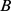 plays (on average) games of smaller size with 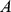 (one two-person game centered on 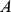 and one four-person game centered on 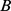) and games of larger size with 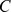 (two four-person games centered at 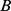 and 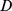, and one three-person game centered on 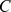). In any case, the replacement graph posited by the graph approach fails to take into account these heterogeneities, since the connections between 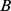 and 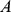 and between 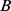 and 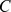 are equally (un)weighted in this graph.

A new modeling framework for studying networked multiplayer games, recently proposed by Gómez Gardeñes and co-workers [60], [61] and further generalized here, is free of these limitations. We call this framework *the bigraph approach*. It consists in (1) defining the interaction bigraph 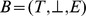 so that top vertices correspond to games and bottom vertices to players, and (2) deriving the replacement graph 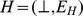 as the (bottom) projection of 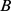 (see Panels C and D of Figure 1). The bottom projection of a bigraph 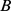 is a graph 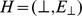 so that 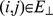 if and only if 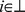 and 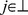 are connected at least once to the same top vertex. In addition to the “unweighted projection” (UP) considered in Ref. [60], we also consider two weighted projections: the “unnormalized weighted projection” (UWP) and the “normalized weighted projection” (NWP). With the UWP method, the weight of the link between two players is proportional to the number of common games played by those players; with the NWP method, the sizes of such groups are taken into account when calculating the weights, so that interactions in smaller groups contribute more to the total weight of the link than interactions in larger groups (see the sec:methods section for details).

The bigraph approach circumvents all of the limitations associated with the graph approach we mentioned above. Since the interaction bigraph is defined at the outset, it can have arbitrary numbers of games and players, different degree distributions for games and players and (if required) relatively low bipartite clustering coefficient. In addition, since the replacement graph is obtained as the bottom projection of the interaction graph, individuals playing together at least one game will be connected in the replacement graph. Hence, the neighborhood of player 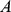 in the replacement graph shown in Panel D of Figure 1 comprises all the individuals 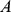 interacts with, i.e. 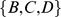. Finally, weighted projections take into account differences in the interaction patterns of players and reflect such differences in the resulting replacement graph. For instance, in the graph shown in Panel D of Figure 1 the weights of the links between players 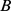 and 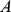 and between players 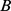 and 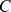 are respectively given by 2 and 3, indicating the number of common games between each pair of players (in Panel D of Figure 1, weights are derived using the UWP method).

In this paper, we make use of the bigraph approach to explore the influence of different topological properties of network structures on the evolutionary dynamics of multiplayer games. We focus on the conventional and the distributed versions of the NPD, as these are among the most studied evolutionary multiplayer games on graphs. Specifically, we investigate the effects of different assumptions on the way of specifying replacement graphs, different top and bottom degree distributions, and different amounts of bipartite clustering. We build interaction bigraphs either from prescribed simple graphs using the graph approach, or from given degree distributions using the configuration model procedure (see the sec:methods section). We denote these interaction bigraphs respectively by the labels fromgraph-X and config-Y–Z, where X stands for the simple graph from which the bigraph is constructed, and Y and Z stand for the degree distributions of the bottom and the top vertices, respectively. Replacement graphs are given either by the graph approach or by the bigraph approach.

## Results

### Replacement Graphs

In the graph approach, the original graph from which the interaction bigraph is constructed automatically determines the replacement graph. As a result, the subset of players involved in imitation/competition with a given individual is generally smaller than the subset of players with whom such individual interacts. This implicit assumption is in stark contrast with most models of two-person games on graphs, where interaction and replacement neighborhoods perfectly overlap. In the following, we present the results of making interaction and replacement neighborhoods coincide in otherwise standard models of evolutionary multiplayer games on graphs.

**Figure 2:**
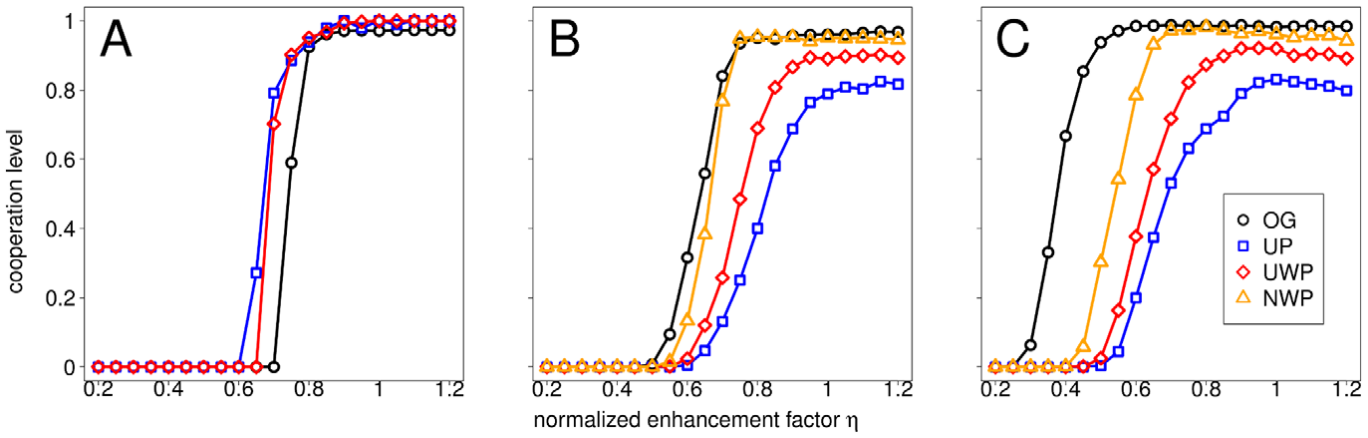
Cooperation level for population structures with different replacement graphs. Results are shown for the conventional NPD (Panels A and B) and the distributed NPD (Panel C). In each case, interaction graphs are constructed following the graph approach from rings (Panel A) or Barabási-Albert scale-free networks (Panels B and C) of order 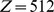 and mean degree 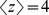. The replacement graph is given by the original graph from which the interaction bigraph is constructed (OG) or the unweighted, unnormalized weighted or normalized weighted projection of the interaction graph (UP, UWP and NWP, respectively).

Figure 2 depicts the results of the evolution of cooperation in the conventional and the distributed NPD for population structures with the same interaction bigraph but different replacement graphs. We plot the cooperation level (the average fraction of Cs for 2000 additional generations after an initial transient of 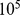 generations) as a function of the normalized enhancement factor 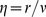, where 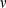 is the average degree of the top nodes of the interaction bigraphs, i.e. 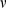 is the mean number of players per game in the population. In each case, the population structure is built (1) by defining a graph 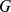 of order 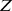 (i.e. 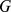 has 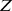 nodes) and mean degree 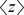, and (2) by constructing the interaction bigraph 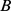 from 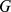 using the graph approach. Hence, 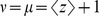, where 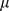 is the average degree of the bottom nodes in the interaction bigraphs, i.e. the mean number of games per player in the population. The replacement graph 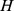 is given either by 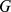 itself (graph approach) or by the projection of 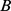 (bigraph approach). In this last case, weights are assigned to the edges of 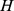 according to one of three methods: UP, UWP and NWP. In any case, individuals engage in a given number of multiplayer games (according to their connectivity in the interaction bigraph) and accumulate payoffs. The accumulated payoff of each player is then associated with its fitness/success, and competition/imitation is implemented by using a finite population analogue of the replicator dynamics [38], [62]: in social learning terms, each individual randomly chooses a neighbor in the replacement graph and, if the neighbor’s success is greater than its own success, it imitates the neighbor’s strategy with a probability proportional to the success difference (see the sec:methods section for details).

Panel A of Figure 2 shows the results for the case where 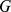 is a ring of degree 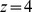. We refer to the resulting bigraph 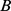 as fromgraph-ring. Referring to the original graph 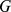, individuals interact with both their first-order neighbors and their second-order neighbors. If the replacement graph is given by the graph approach, only first-order neighbors in 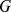 are considered for competition/imitation. If the replacement graph is given by the bigraph approach, both first-order and second-order neighbors in 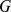 are considered for competition/imitation, possibly with a probability depending on the number of common games (UWP). Note that the larger replacement neighborhoods due to the bigraph approach favors cooperation slightly, but systematically. A detailed analysis of the origin of such promotion, considering the case of two contiguous clusters of Ds and Cs in a ring of degree 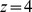, can be found in section 1 of Text S1 and Figure S1.

While the larger replacement neighborhoods brought about by the bigraph approach are beneficial to cooperation in bigraphs constructed from rings, they are detrimental to cooperation in bigraphs constructed from Barabási-Albert (BA) scale-free networks, which we call fromgraph-ba. Indeed, as evidenced in Panels B and C of Figure 2, in this case there is systematically less cooperation if replacement neighborhoods coincide with interaction neighborhoods (bigraph approach) than if the original graph is taken as the replacement graph (graph approach). Additionally, in the former case the assignment of weights to the edges of the replacement graph plays a key role in BA networks, as it is evident from the ordering of the curves, with NWP leading to more cooperation than UWP, and UWP to more cooperation than UP.

In order to explain these results, let us briefly recall the mechanism responsible for the promotion of cooperation in the distributed NPD when the interaction and replacement graphs are derived from scale-free networks using the graph approach [38]. Scale-free networks are characterized by the co-existence of few hubs (very well connected individuals) with a vast majority of leaves (poorly connected individuals). Due to their large connectivity, hubs not only take part in many games, consequently accumulating high payoffs, but are also often targeted for competition/imitation by their neighbors. As a result of these two factors, C-hubs and D-hubs easily spread their strategies to their less connected neighbors. However, while C-hubs are favored by a positive feedback mechanism (the more they are imitated, the more Cs in their neighborhoods, and the more their own accumulated payoffs increase) D-hubs are penalized by a negative feedback mechanism (the more they are imitated, the more Ds in his neighborhood, and the more their own accumulated payoffs decrease) that eventually leads to their own demise. Hubs’ inherent success along with the feedback mechanisms favoring Cs in inter-hub competition have been studied using star and double-star graphs as simple models of connectivity patterns in scale-free networks [38], [39].

**Figure 3:**
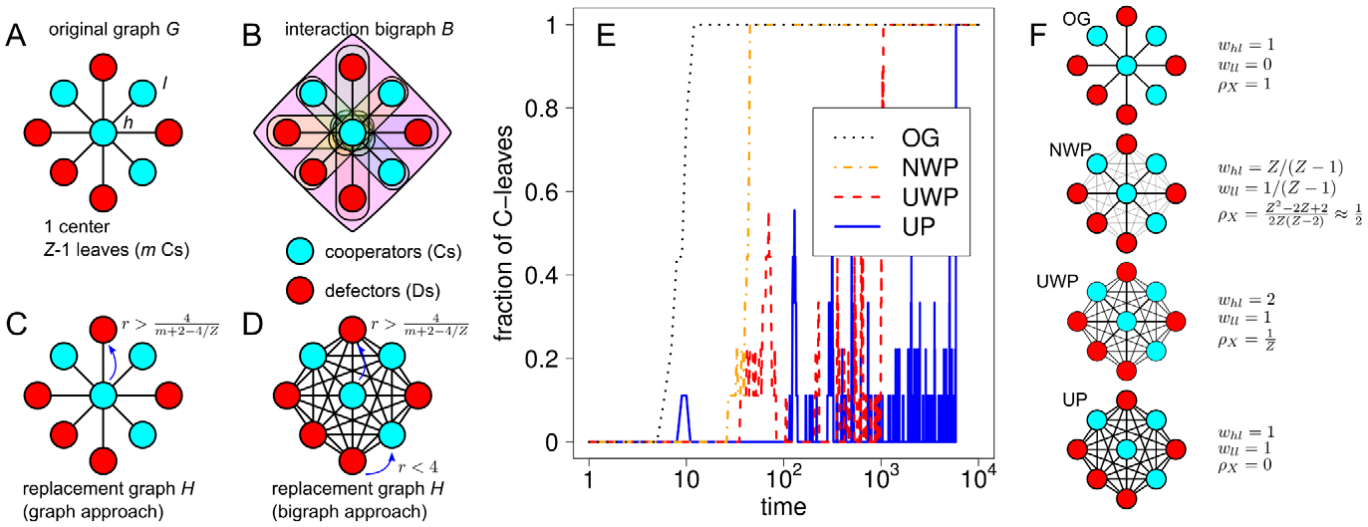
Evolutionary dynamics on stars. Consider a star graph 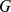 (Panel A) consisting of one C-center connected to 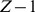 leaves (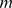 of which are Cs) and the resulting interaction hypergraph 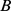 (Panel B) constructed from 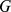 using the graph approach. We assume that social interactions are modeled by the distributed NPD. In the graph approach, 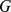 is taken as the replacement graph (Panel C). In this case, competition/imitation occurs only between the center and the leaves. The C-center invades D-leaves for values of 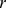 above a critical value which reduces to 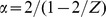 if 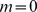 (the C-center is the only C). In the bigraph approach, the replacement graph is given by the projection of the interaction bigraph, so that leaves are now interconnected and the resulting topology is no longer a star but a complete graph (Panel D). The creation of these new links allows for inter-leaf competition/imitation, which is favorable to Ds if 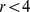. As a result, for 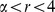, the time to fixation to the absorbing state where all individuals are Cs can become arbitrarily large depending on the weights attached to the links of the replacement graph, as it is shown in Panel E for 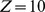, 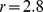 and for replacement graphs given by the OG, NWP, UWP and the UP methods. Panel F shows these replacement graphs together with the values of the weights of the links (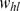 for the weight of the link between the center and a leaf; 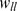 for the weight of the link between two leaves) and their centralization indices (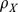). Note that more centralized graphs correspond to those more favorable to the spreading of cooperative behavior from the center. See section 2 of Text S1 for the derivation of the invasion conditions shown in Panels C and D, and sections 4 and 5 of Text S1 for the derivation of the centralization indices shown in Panel F.

**Figure 4:**
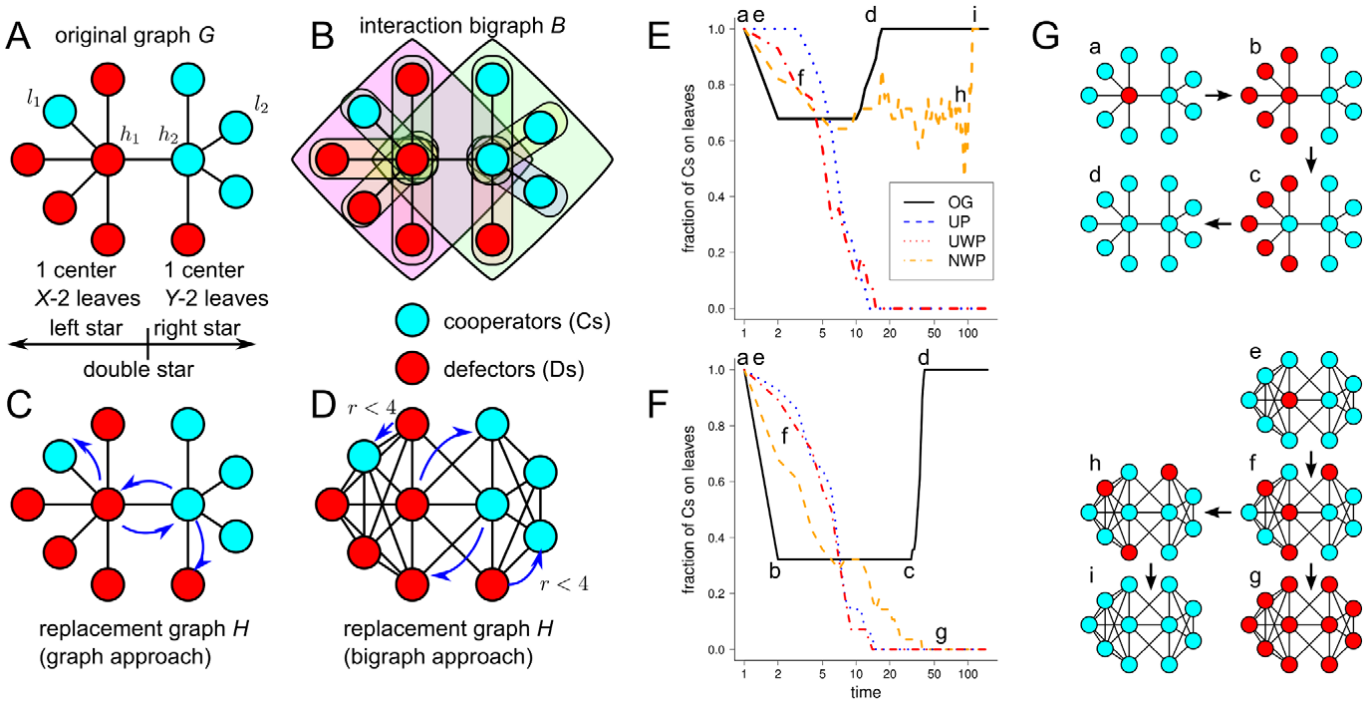
Evolutionary dynamics on double stars. Consider a double-star graph 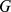 (Panel A) consisting of a left and a right star connected by the centers, and the resulting interaction hypergraph 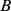 (Panel B) constructed following the graph approach. In the graph approach, 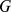 is taken as the replacement graph (Panel C). In this case, and for a wide range of values of 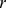, spreading occurs preferentially from the centers (or hubs) to their respective leaves. Long-term evolution will ultimately depend on inter-hub competition, which is favorable to C-hubs due to the positive and negative feedback mechanisms brought about by the spreading from centers to (own) leaves. In the bigraph approach, the replacement graph is given by the projection of the interaction bigraph (Panel D), so that the center of one start gets connected to the leaves of the other star and leaves of the same star get connected with each other. This not only allows successful centers to breed copies of themselves in the leaves of the other star, but also makes inter-leaf competition possible, which is favorable to Ds if 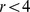. As a result, the feedback mechanisms on which the evolution of cooperation on heterogeneous graphs is based are diminished and the evolutionary outcome is more favorable to Ds. This is illustrated in Panels E and F, which show typical scenarios for the time evolution of the fraction of Cs under the distributed NPD (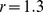) on the leaves of double-star graphs (Panel E: 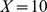, 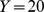; Panel F: 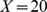, 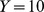), for replacement graphs given by the OG, UP, UWP and NWP methods. In all cases we placed Cs on all nodes of the double-star, except for the left center, where we placed a D (see configurations **a** and **e** of Panel G). If the replacement graph is given by the original graph (OG), the dynamics are such that, typically, the D-center invades the leaves of his star (configuration **b**), then the C-center invades the D-center (**c**) and finally D-leaves on the left star are invaded by the C-center (**d**). When the replacement graph is given by the projection of the interaction graph, Ds can now easily spread from the initial center until they invade the whole population (**e**, **f**, **g**). Weights attached to the links of the projection play a key role in this case, with NWP still favoring Cs when the connectivity of the left star is small compared to that of the second star (**e**, **f**, **h**, **i**). See section 3 of Text S1 for the analytical derivation of the results shown in this figure.

If the replacement graph 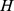 is no longer the original graph 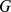 (graph approach) but it is rather assumed to be the projection of the interaction bigraph 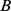 (bigraph approach), many additional links are present in 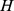 that were not in 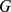. Indeed, since each top node of degree 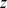 induces a clique consisting of 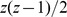 edges, the projection of 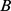 is a relatively dense graph, particularly if the top degree distribution is highly heterogeneous [63]. This higher density of the replacement graph is at the origin of the hindering of the evolution of cooperation when moving from the graph approach (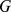 taken as 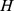) to the bigraph approach (the projection of 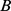 taken as 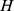). Figures 3 and 4 show this effect for bigraphs built according to the graph approach from star and double-star graphs. In stars, and when the replacement graph is given by the projection of the interaction bigraph, leaves get connected to each other so that 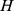 is now a complete graph (see Panel D of Figure 3). This hinders the spreading of cooperative behavior from a C-center when defective leaves earn a higher payoff than cooperative leaves. In double-star graphs, leaves of the same star get interconnected and the center of one star gets connected to the leaves of the other star (see Panel D of Figure 4). This increased interconnection hinders cooperation by partially destroying both the positive feedback around C-centers and the negative feedback around D-centers on which inter-hub competition is based in the model of Ref. [38]. Note that, in all cases, the magnitude of these unfavorable effects depends on the weights attached to the links of the replacement graph. Indeed, different projection methods lead to different weight distributions, which in turn affect the topological importance of different nodes in the evolutionary process. Such topological importance can be captured by what we call in this paper *replacement centrality*, which we define as the expected number of times a given node/individual is selected for competition/imitation by its neighbors (see section 4 of Text S1). Other things being equal, nodes with a higher replacement centrality play a more influential role in the evolutionary dynamics. We find that the level of centralization of the replacement graph (defined as the degree to which a single node is more central than others in the network; see section 4 of Text S1) correlates with the amount of cooperation exhibited in these topologies, as measured by the inverse fixation time of a single C-center in a star graph (see Panel E of Figure 3) or by the cooperation level in BA scale-free networks (Panels B and C of Figure 2). Figure S2 shows that the relationship between projection method and centralization of the network found in star graphs (OG 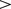 NWP 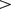 WP 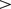 UP) is maintained in BA scale-free networks. As evidenced by Figure S2 and Panels B and C of Figure 2, weight distributions leading to more centralized replacement graphs are also responsible for higher cooperation levels.

### Degree Distributions

In bigraphs constructed using the graph approach, *group diversity* (heterogeneity in the number of players per game) is inextricably intertwined with *individual diversity* (heterogeneity in the number of games per player). Indeed, the top degree distribution (determining group diversity) is exactly the same as the bottom degree distribution (determining individual diversity) in bigraphs built using the graph approach. In order to analyze group diversity and individual diversity independently of each other, we made use of random configuration model bigraphs (for which the degree sequences of top and bottom vertices can be specified independently of each other) as interaction bigraphs. We used two different degree sequences for top and bottom vertices: a constant sequence (all degrees are the same) and the degree sequence of a BA scale-free graph, which approximately follows a power-law. Combinations of these two degree sequences resulted in four bigraphs: config-reg-reg (with homogeneous top and bottom degree distributions), config-ba-reg (with heterogeneous bottom and homogeneous top degree distributions), config-reg-ba (with homogeneous bottom and heterogeneous top degree distributions), and config-ba-ba (with heterogeneous bottom and top degree distributions). The reason for using the degree sequence of a BA graph instead of determining the degree sequence by another method (for instance, by sampling the sequence from a random variable distributed according to a power-law distribution) is to be able to compare the results obtained for config-ba-ba with those obtained for fromgraph-ba in the subsec:replacement subsection. Indeed, config-ba-ba has the same top and bottom degree sequences as fromgraph-ba, and can be effectively thought of as a randomization of such network.

**Figure 5:**
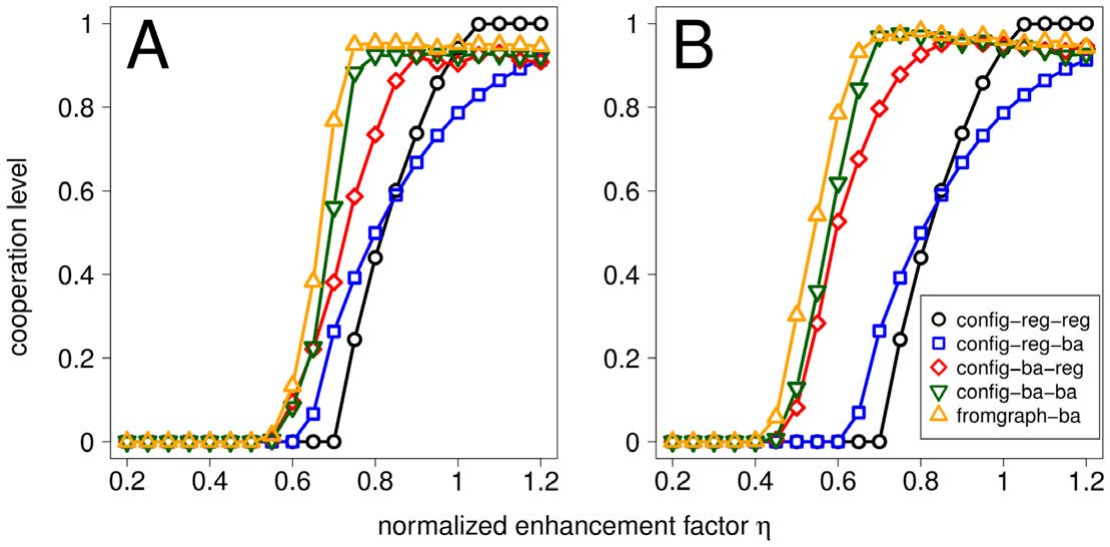
Cooperation level for population structures with different degree distributions. Panel A shows results for the conventional NPD; Panel B for the distributed NPD. config-X–Y stands for a configuration model bigraph with a degree sequence of type X for the bottom vertices (players) and of type Y for the top vertices (games). For the degree sequences themselves, reg is a regular sequence and ba is the degree sequence of a Barabási-Albert network. A bigraph constructed from a Barabási-Albert network following the graph approach (fromgraph-ba) is shown for comparison purposes. Parameters: 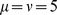, 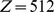, replacement graph given by the NWP method.

Figure 5 shows the results for the evolution of cooperation in the conventional and the distributed NPD for the four configuration model bigraphs and for the fromgraph-ba. Let us consider first the results for config-reg-reg, i.e. the homogeneous population structure lacking social diversity of any kind. As shown in the figure, this network is able to sustain cooperation for values of 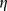 above 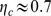. Furthermore, cooperation is fully established for 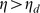, with 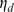 close to its value for infinite well-mixed populations (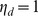). For 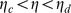, Cs and Ds co-exist in dynamical equilibrium. If group diversity is introduced (config-reg-ba), the co-existence zone grows so that 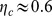, and 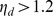. This shows that group diversity has mixed effects in the evolutionary dynamics, promoting cooperation (with respect to config-reg-reg) up to a critical value 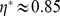, and hindering cooperation above this value. If diversity is instead introduced at the individual level (config-ba-reg), cooperation is evolutionarily viable for 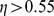 in the conventional NPD and for 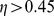 in the distributed NPD. Note, nonetheless, that defective behavior is not completely eradicated, not even for 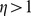. From these results, it is evident that individual diversity leads to higher cooperation levels than group diversity (compare the curves for config-ba-reg with those for config-reg-ba) for all values of 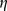. We also note that the levels of cooperation slightly improve when both kinds of social diversity are simultaneously present (compare config-ba-ba to config-ba-reg and config-reg-ba). Finally, the results obtained with config-ba-ba are almost the same as those obtained with fromgraph-ba, which suggests that the higher topological correlations present in fromgraph-ba and absent in config-ba-ba play a rather small role in the evolutionary dynamics.

The results for networks with homogeneous bottom degree distributions (config-reg-reg and config-reg-ba) and for networks with heterogeneous bottom degree distributions (config-ba-reg, config-ba-ba and fromgraph-ba-ba) differ not only quantitatively in their cooperation levels, but also qualitatively in their dynamics. Indeed, intermediate cooperation levels for bigraphs with homogeneous bottom degree distributions are mostly due to the co-existence of Cs and Ds. Contrastingly, in the case of bigraphs with heterogeneous bottom degree distributions intermediate cooperation levels are due to bi-stability, so that the vast majority of times the dynamics reaches the absorbing states of full defection or full cooperation. In this last case, intermediate cooperation levels are almost entirely determined by the proportion of times the dynamics ended up in the full cooperation absorbing state.

**Figure 6:**
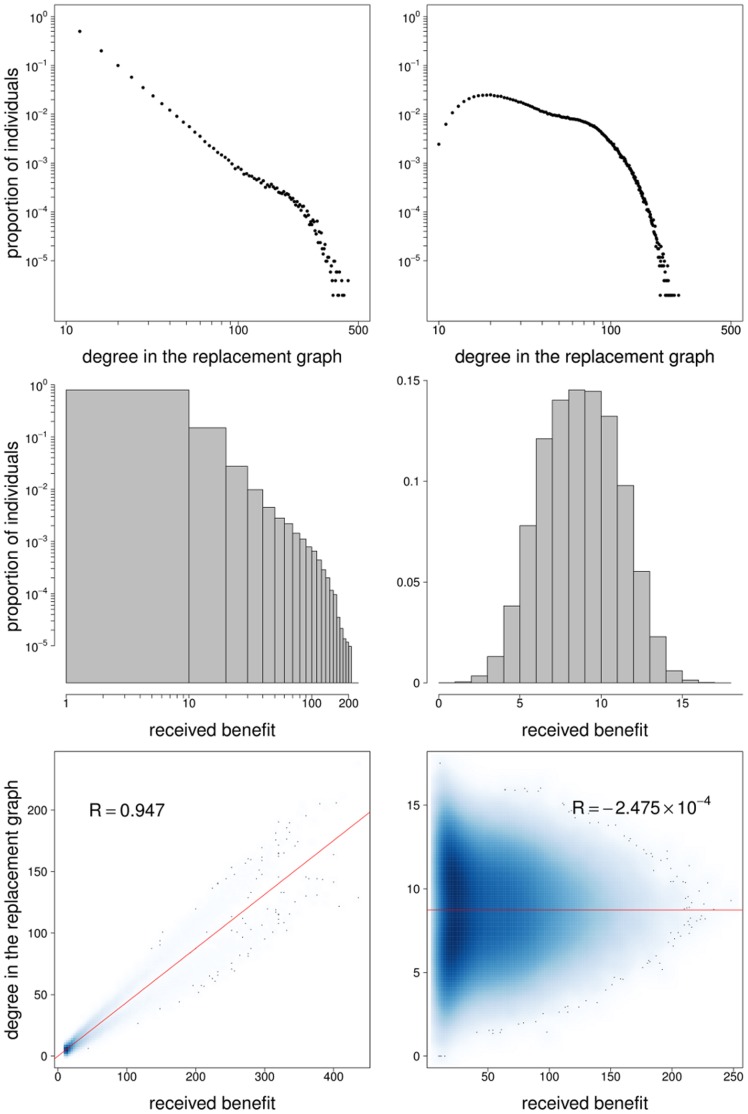
Statistics for config-ba-reg and config-reg-ba. The figure shows some statistics for config-ba-reg (left panels) and config-reg-ba (right panels). Top panels: degree distribution of the replacement graph. Middle panels: histograms for the received benefit. The received benefit is calculated as the payoff for Ds when approximately half of the population are Cs (randomly distributed) under the distributed NPD. Bottom panels: smooth scatter plots, regression lines and Pearson’s correlation coefficients for the received benefit vs. degree in the replacement graph. Parameters: 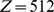, 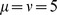 and 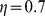. The figures show statistics for 100 randomly generated networks of each type.

Figure 6 provides some insight on the different results obtained when diversity is introduced at the individual level (config-ba-reg) or at the group level (config-reg-ba). First, note that the degree distribution of the replacement graph for config-ba-reg is highly heterogeneous (see top panels of Figure 6). Indeed, it is well known that the degree distribution of the projection of a bigraph with a power law bottom degree distribution also follows a power law [64]–[66]. Contrastingly, the degree distribution of the replacement graph for config-ba-reg is less heterogeneous. A second important difference between config-ba-reg and config-reg-ba is the way received benefits are distributed on these networks. When the population consists of 50% Cs randomly placed on the bottom vertices of the bigraph, the distribution of received benefits closely follows a power-law in the case of config-ba-reg, but it approximately follows a normal distribution in the case of config-reg-ba (see middle panels of Figure 6). The reason behind these different distributions is that on config-ba-reg both the per-capita per-game contribution and the number of games per individual are highly variable while on config-reg-ba they are constant. This leads to a highly heterogeneous distribution of received benefits on config-reg-ba and to a relatively homogeneous distribution on config-reg-ba. Finally, while there is a strong correlation between connectivity in the replacement graph and received benefit in config-ba-reg, such correlation is practically inexistent in config-reg-ba (see bottom panels of Figure 6). Indeed, for config-ba-reg hubs in the replacement graph are individuals participating in many games and hence accumulating large payoffs. Contrastingly, for config-reg-ba highly connected individuals in the replacement graphs are those participating in large groups, which have on average the same *proportion* of Cs and hence produce the same amount of public good than smaller groups. As a result, the evolutionary dynamics on config-ba-reg is dominated by a small number of very well connected and powerful individuals, while config-reg-ba is far more homogeneous, both concerning connectivity in the replacement graph and accumulated payoffs. These differences translate into two different modes of evolution. In config-ba-reg (see Figure S3) the influence of hubs is decisive to the evolutionary outcome, so that a majority of C-hubs leads the whole population to the all-Cs absorbing state, while a majority of D-hubs leads the population to the all-Ds absorbing state. Additionally, the proportion of Cs is also higher in high-degree classes (very well connected individuals) than in low-degree classes (poorly connected individuals). Contrastingly, in config-reg-ba (see Figure S4) the evolutionary dynamics is largely independent of what happens with well-connected individuals, and evolution unfolds as a process of dynamical self-organization in which Cs tend to cluster in small groups which are more favorable to cooperation while Ds tend to do so in large groups which are more favorable to defection.

### Bipartite Clustering Coefficient

The bipartite clustering coefficient captures the degree to which bottom vertices’ neighborhoods overlap (see section 6 of Text S1 for details). As pointed out in the sec:introduction section, interaction bigraphs built using the graph approach lead, by construction, to relatively high bipartite clustering coefficients. In order to assess the real importance of clustering in the evolutionary dynamics, we considered four interaction bigraphs with the same top and bottom degree distributions (regular sequences in all cases) but different bipartite clustering coefficients: fromgraph-ring (constructed from a ring network of degree 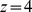), fromgraph-reg (constructed from a random regular network of degree 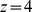), fromgraph-vn (constructed from a square lattice with a von Neumann neighborhood), and config-reg-reg (random configuration model with regular top and bottom degree sequences).

**Figure 7:**
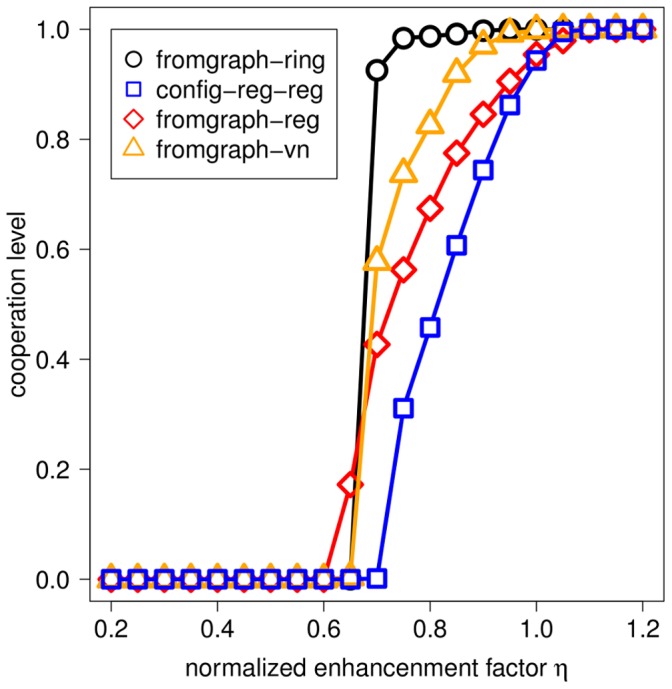
Cooperation level for bigraphs with different bipartite clustering coefficients. The interaction bigraphs are constructed following the graph approach with a ring (fromgraph-ring), a square lattice with von Neumann neighborhoods (fromgraph-vn), or a regular random network (fromgraph-reg) of degree 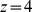 as original graphs, or given by a configuration model bigraph with regular degree sequences for both top and bottom vertices (config-reg-reg). In all four cases the degree distributions of top and bottom vertices is a regular sequence with 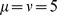, the replacement graph is given by the normalized weighted projection (NWP) of the interaction bigraph, and 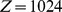.

**Figure 8:**
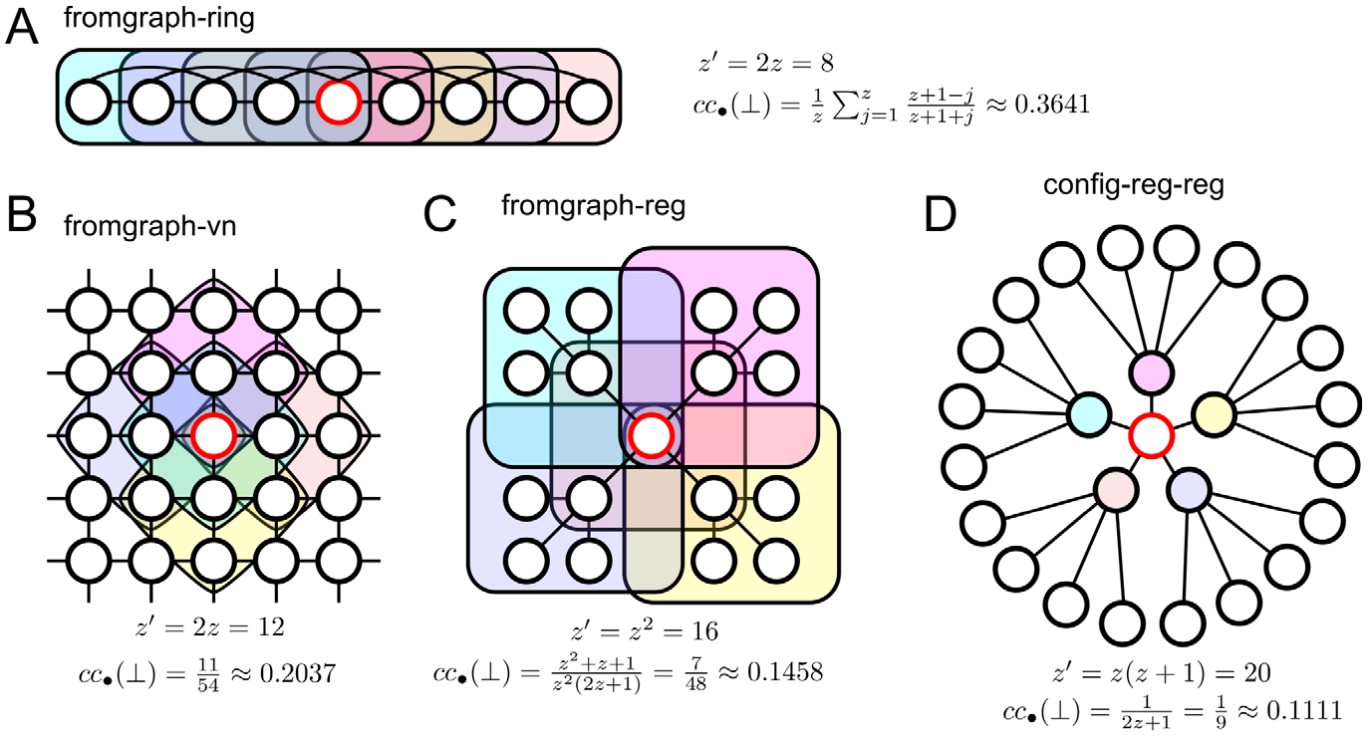
Graphical representation and bipartite clustering coefficients of different interaction bigraphs. The figure shows typical interaction neighborhoods for a focal individual (red node) as well as the degree of the replacement graph (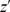) and the bipartite clustering coefficient (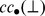) for fromgraph-ring (Panel A), fromgraph-vn (Panel B), fromgraph-reg (Panel C) and config-reg-reg (Panel D). For all networks, 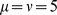. Values of 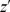 and 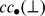 are exact for fromgraph-ring and fromgraph-vn and analytical approximations (assuming networks are Bethe lattices) for fromgraph-reg and config-reg-reg.

Figure 7 shows the cooperation levels under the conventional NPD and Figure 8 the bipartite clustering coefficient and the mean degree of the replacement graph for these different bigraphs. Interestingly, bigraphs with more bipartite clustering (and consequently lower mean degree in the replacement graph) lead in general to equal or higher cooperation levels for all the considered values of the normalized enhancement parameter 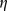. These results make sense in the light of well established results on the effects of local interactions on the evolutionary dynamics of the pairwise and multiplayer versions of the NPD. It is well known that spatial structure enables Cs to form clusters within which they preferentially interact with other Cs, thus reducing the exploitation by surrounding Ds. Cluster formation is brought about by a feedback mechanism resulting from imitation/competition with direct neighbors that amplifies initial inhomogeneities in the distribution of strategies. As it is shown in Figure S5, large values of bipartite clustering coefficient favor cluster formation by allowing Cs to find each other more easily and to reduce the number of connections with surrounding Ds.

## Discussion

Since the seminal works by Axelrod [67] and Nowak and May [9] on the evolution of cooperation on lattices, games on graphs have traditionally made use of unipartite graphs in order to model population structures. Despite its usefulness for exploring the effects of local interactions on the evolutionary dynamics of two-player games, the use of unipartite graphs as population structures entails a certain number of construction limitations when applied to general multiplayer games, leading not only to a lack of flexibility but also to unrealistic assumptions about the topological properties of networked populations. In this paper, we have shown how the use of bipartite graphs and of constructing procedures that fully take into account the bipartite nature of social and biological populations can circumvent the limitations of the standard graph approach, opening up new opportunities for studying the role of different properties of network topologies on the evolution of strategic interactions. In particular, it is important to emphasize the need of explicitly defining two graphs: the interaction bigraph, determining who plays with whom, and the replacement graph, determining who competes with whom. As demonstrated in this paper, different ways of constructing any of these two graphs or of deriving one from the other can have important consequences in the evolutionary dynamics of multiplayer games.

First, the implicit assumption that the replacement graph coincides with the original graph in the graph approach is crucial for the success of BA scale-free networks as cooperation-promoting topologies reported in Ref. [38]. When the replacement graph is derived in a more natural way, so that interaction and replacement neighborhoods perfectly overlap (the usual assumption in evolutionary two-person games on networks) cooperation is hindered in BA scale-free networks to a point that any advantage of social heterogeneity is effectively canceled by the resulting larger replacement neighborhoods (see Figure 2). The introduction of weights in the replacement graph somewhat alleviates this problem, as weighted links partly restore the high centralization characteristic of BA scale-free networks.

Second, while individual diversity (heterogeneous bottom degree distributions) systematically fosters cooperation, group diversity (heterogeneous top degree distributions) promotes cooperation up to a critical value of the enhancement factor, but hinders cooperation above such value (see Figure 5). We also showed that networks with both kinds of social diversity foster more cooperation than networks with only one kind of diversity, but that the difference between the cooperation levels of networks with both individual and group diversity and the cooperation levels of networks with only individual diversity are relatively small. Finally, intermediate cooperation levels in networks without individual diversity are mostly due to co-existence of Ds and Cs, while intermediate cooperation levels in networks with individual diversity are characterized by bi-stable evolutionary dynamics. In other words, the results for config-reg-reg and config-reg-ba shown in Figure 5 can be better understood as representing the final proportion of Cs in a population where both Cs and Ds are present. Contrastingly, the results for config-ba-ba, config-ba-reg and fromgraph-ba can be better interpreted as a probability of ending up in a fully cooperative state when starting from a condition where 50% Cs are randomly placed on the network.

Third, bipartite clustering, i.e. group overlap, plays an important role in the evolution of cooperation under the conventional NPD. We provided clear evidence of the beneficial role of bipartite clustering on cluster formation and, consequently, on the evolution of cooperation on regular structures. In this respect, our results mirror similar conclusions on the beneficial effects of unipartite clustering on the evolution of cooperation under the standard evolutionary two-player PD [68]–[70].

Apart from the present paper and to the best of our knowledge, only two studies have made use of the bigraph approach for studying evolutionary multiplayer games: Ref. [60], where the use of bigraphs as population structures for evolutionary games on networks was first introduced, and Ref. [61], a subsequent study on the effects of social diversity on the evolution of cooperation under the NPD. In the first of these studies, the evolution of cooperation under the NPD on a real bipartite collaboration network is compared to the dynamics on its bottom projection. Higher cooperation levels are found for the bipartite network than for its projection. These results have been interpreted as hinting that “the intrinsic group structure (described by means of the bipartite graph) promotes cooperation in PGGs, this being a new mechanism for this phenomenon beyond the scale-free character and other features of the one-mode (projected) complex network” [60]. We would like to point out that a simpler explanation is that, by construction, the mean group size in the bigraph built from a projected network is always larger than the mean group size in the original bipartite network, and that larger group sizes hinder the evolution of cooperation under the NPD. In order to assess the influence of group structure and other mesoscopic properties on the evolutionary dynamics, a comparison of real bipartite networks with their “randomized” versions should be carried out, as it has been done for real unipartite networks and two-person games [71].

In the second study (Ref. [61]) the evolutionary dynamics of the conventional and distributed versions of the NPD were investigated on interaction bigraphs with tunable individual diversity but no group diversity at all. The main finding of this study is that bigraphs with low individual diversity (Poisson-like bottom degree distributions) can actually allow for more cooperation than bigraphs with high individual diversity (bottom degree distributions following a power law) in the case of the conventional NPD. This result contrasts sharply with our own results, which suggest that individual diversity generally promotes cooperation. Note, however, that we used both a different network model (configuration random networks) and different degree distributions (with zero instead of moderate individual diversity). These different setups could account for the divergent results. We also note that Gómez-Gardeñes et al. [61] suggest that the ability of BA scale-free networks to outperform homogeneous networks reported in Ref. [38] is “intrinsically due to the entanglement of social and group heterogeneities”. Although our own results partially support this view, given the (moderate) synergy between individual and group diversity, we have provided evidence that the promotion of cooperation reported in Ref. [38] is mainly due to the implicit assumption that the replacement graph is equal to the original graph from which the interaction topology is constructed.

The choice of the NPD as case of study in this paper was based on the fact that most of the theoretical work on evolutionary multiplayer games has focused on this particular game. However, recent empirical [29] and theoretical [72] work testifies a growing discomfort with the NPD as model of realistic social dilemmas, in particular because of its linearity and because of the fact that cooperation is a strictly dominated strategy in this game. Several of the conclusions drawn in the present study will necessarily change if strategic interactions are modeled after PGGs different from the NPD. For instance, it has been recently shown that, even in the absence of a fixed topology, group diversity can importantly affect the evolutionary dynamics of non-linear PGGs [73]. In the light of these results, we would expect group diversity to play a more prominent role in the evolutionary dynamics of non-linear games played on bigraphs with highly heterogeneous top degree distributions. Also, bipartite clustering could be partially detrimental, instead of largely beneficial, for the evolution of cooperation if the social dilemma is modeled after a multiplayer game with a structure similar to the snowdrift game, as it is already the case for two-person games [62].

## Methods

### Population Structures

Population structures are modeled by means of two graphs: the interaction bigraph 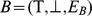 and the replacement graph 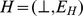. The two sets of vertices of the interaction bigraph (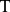 and 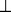) represent, respectively, the set of groups/games and the set of individuals/players.

#### Graph approach

In what we call the graph approach [37], [38], first the replacement graph 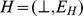 is defined, then the interaction bigraph 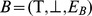 is constructed from the replacement graph as follows. Denote by 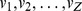 the vertices of the graph 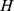 and by 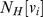 the closed neighborhood of vertex 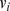, defined as the set of vertices adjacent to 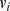 plus 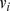 itself. Further, denote by 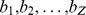 the bottom (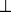) vertices of 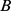 and by 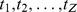 the top (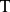) vertices of 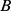. Then, 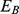 is defined as the set of all pairs 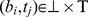 such that 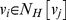.

#### Bigraph approach

In what we call the bigraph approach [60], first the interaction bigraph 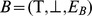 is defined, then the replacement graph 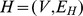 is constructed by projecting the interaction bigraph into its set of bottom vertices. In addition, weights can be attached or not to the edges of 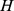 according to one of the following three methods:

1. **Unweighted projection (UP).** As done in [60], no weights are attached to the edges or, equivalently, the weights of all edges have a value of one.
2. **Unnormalized weighted projection (UWP).** The weight 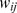 of the link 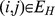 is given by the number of games 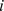 and 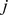 are connected to in the interaction bigraph [55]. From a social learning perspective, the reason behind this heuristic is that the more often 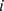 interacts with 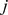, the better 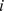 is supposed to be acquainted with 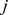 and therefore the more often 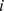 should consider 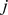 as target for imitation.
3. **Normalized weighted projection (NWP).** The weight 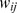 is given by [56]

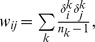
where 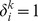 if 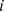 participates in game 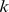, 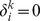 otherwise, and 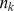 is the number of players of game 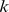. From a social learning perspective, the reason behind this heuristic is the assumption that individuals get acquainted with others more easily in smaller than in larger groups.

#### Bigraphs built from simple graphs using the graph approach

For fromgraph-X interaction bigraphs, we considered four different kinds of graphs: rings, scale-free networks, square lattices with von Neumann neighborhoods and regular random networks. Rings are one-dimensional lattices with degree 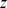. Regular random networks (maximally random graphs where each node has the same degree 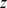) were constructed using the igraph [74] implementation of the algorithm by Viger and Latapy [75]. Scale-free networks were obtained by means of the Barabási-Albert (BA) model [18], i.e. growing networks using a preferential attachment rule. In order to get graphs with average degrees exactly equal to 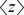, we started the growing procedure from a fully connected graph of 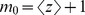 nodes, and added 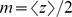 new edges per new node.

#### Configuration model bigraphs

Config-X–Y bigraphs were constructed using the configuration model [63], [64], [76] with a top degree distribution of type X and a bottom degree distribution of type Y. For the degree distributions, we used regular sequences (reg) and degree sequences from BA scale-free networks (ba), constructed following the procedure mentioned before.

### Multiplayer Games

Each individual 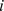 participates in all games 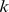 such that 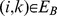. The social success of an individual is given by the sum of the payoffs obtained in all games it takes part in. We considered two versions of the NPD: the conventional NPD and the distributed NPD [38], [39]. In the conventional NPD, the payoffs of a D and a C in a group 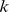 of size 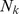 are respectively given by 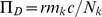 and 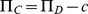, where 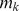 is the number of Cs in group 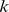, 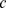 is the cost of cooperation and 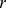 is the enhancement factor. In the distributed NPD, each C of degree 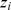 (i.e. taking part in 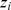 games) contributes 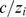 to each game, so that the overall contribution of any C is equal to 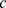. In this case, the payoff of individual 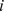 with strategy 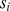 (1 if C, 0 if D) is given by [38]

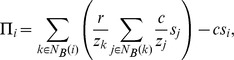
where 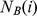 is the open neighborhood of player 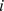 in 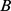 (i.e. the set of games played by 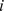), 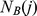 is the open neighborhood of game 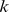 in 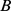 (i.e. the set of players participating in game 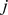), and 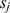 and 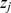 stand respectively for the strategy and the degree of the 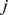-th player in the 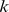-th group.

### Evolutionary Dynamics

The success/fitness of each individual was calculated as the sum of the payoffs obtained in all the games it participates in. Strategies are updated synchronously using a finite population analogue of the replicator dynamics commonly used in the literature of games on networks [38], [62]. When updating the strategy of individual 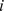, a neighbor 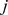 of 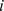 in the replacement graph is randomly chosen with a probability 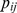 given by
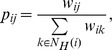
where 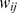 is the weight of the link 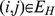. Denote by 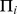 the accumulated payoff of individual 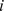. Then, if 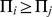, 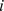 stays with its current strategy; otherwise it changes its strategy to 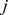
’s with a probability given by 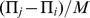, where 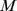 is a normalization factor given by the highest possible difference between the accumulated payoffs of 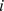 and 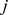.

### Simulations

Simulations were started with 50% of Cs randomly placed on the graph. We measured the average fraction of Cs for 2000 additional generations after an initial transient of 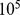 generations, and called this value the cooperation level. Data points in Figures 2 and 5 correspond to the mean cooperation level over 1000 simulations; data points in Figure 7 correspond to the mean cooperation level over 100 simulations. A new realization of the graph is done for each simulation.

## Supporting Information

Figure S1
**Evolutionary dynamics on rings.** In the inset, we plot a ring of degree 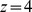: the neighborhood of each node comprises the closest two nodes to the left and to the right. Following the graph approach, each node is the center of a game of size five so that each individual ends up interacting with the closest four neighbors to the left and the closest four neighbors to the right. We assume that the initial distribution of strategies is such that nodes 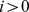 are Cs and nodes 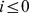 are Ds. In the main panel, we plot the probabilities of switching strategies for the individuals at the boundary (nodes 0 and 1) when the replacement graph is given by the original graph (OG) and when it is given by the unweighted projection (UP) of the interaction bigraph. As shown, 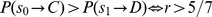 for the graph approach, while 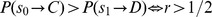 for the bigraph approach. See section 1 of Text S1 for the calculation of these probabilities.(TIFF)

Figure S2
**Centralization of the replacement graphs for interaction bigraphs built from Barabási-Albert scale-free networks.** Each boxplot shows the distribution of the centralization for a random sample of 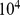 replacement graphs given by the original graph (OG), the normalized weighted projection (NWP), the unnormalized weighted projection (UWP) and the unweighted projection (UP). In all cases, the original graph is a Barabási-Albert scale-free network of order 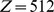 and mean degree 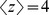. The projections are taken from bipartite graphs constructed from the original graph using the graph approach. Notice that more centralized networks lead to higher cooperation levels in Panels B and C of Figure 2 in the main text. See section 4 of Text S1 for the definition of the centralization indices used in this figure.(TIFF)

Figure S3
**Time-dependence of the fraction of cooperators for different connectivity classes in the config-ba-reg network.** The figure shows the fraction of Cs among low-degree (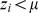), medium-degree (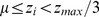) and high-degree (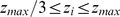) individuals, for two different simulation runs. In Panel A, initially more than the 60% of the highly-connected individuals are Cs. C-hubs lead the evolutionary process and diffuse cooperative behavior among their less connected neighbors. In Panel B, initially less than 40% of the hubs are Cs. Less connected individuals quickly turn to defection, with medium-degree and high-degree individuals eventually following the trend. Parameters: 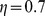, 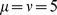 and 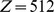.(TIFF)

Figure S4
**Time-dependence of the average experienced group size and of the fraction of cooperators in groups of different size for config-reg-ba.** The figure shows the mean experienced group size for Cs and Ds (top panels) and the fraction of Cs in small (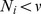), medium-sized (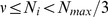) and large (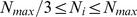) groups (bottom panels) for 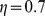 (left panels) and 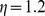 (right panels). The evolutionary dynamics on this population structure is such that Cs preferentially cluster together in small groups and Ds cluster together in large groups. Parameters: 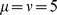 and 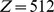.(TIFF)

Figure S5 **Time evolution of the degree of assortment in the replacement graphs of interaction bigraphs with different bipartite clustering coefficients.** The figure shows the time evolution of the degree of assortment in the replacement graph. See section 7 of Text S1 for the definition of degree of assortment we used in this figure.
(TIFF)

Text S1 **Supporting Text on analysis of the evolutionary dynamics on rings, stars and double-star graphs, and on definitions of replacement centrality, centralization, bipartite clustering coefficient and degree of assortment.**
(PDF)
