# Supplementary figures and images for: Bipartite Graphs as Models of Population Structures in Evolutionary Multiplayer Games
Source: PLoS One. 2012 Sep 10;7(9):e44514. doi: 10.1371/journal.pone.0044514 (PMC3438187; doi:10.1371/journal.pone.0044514)

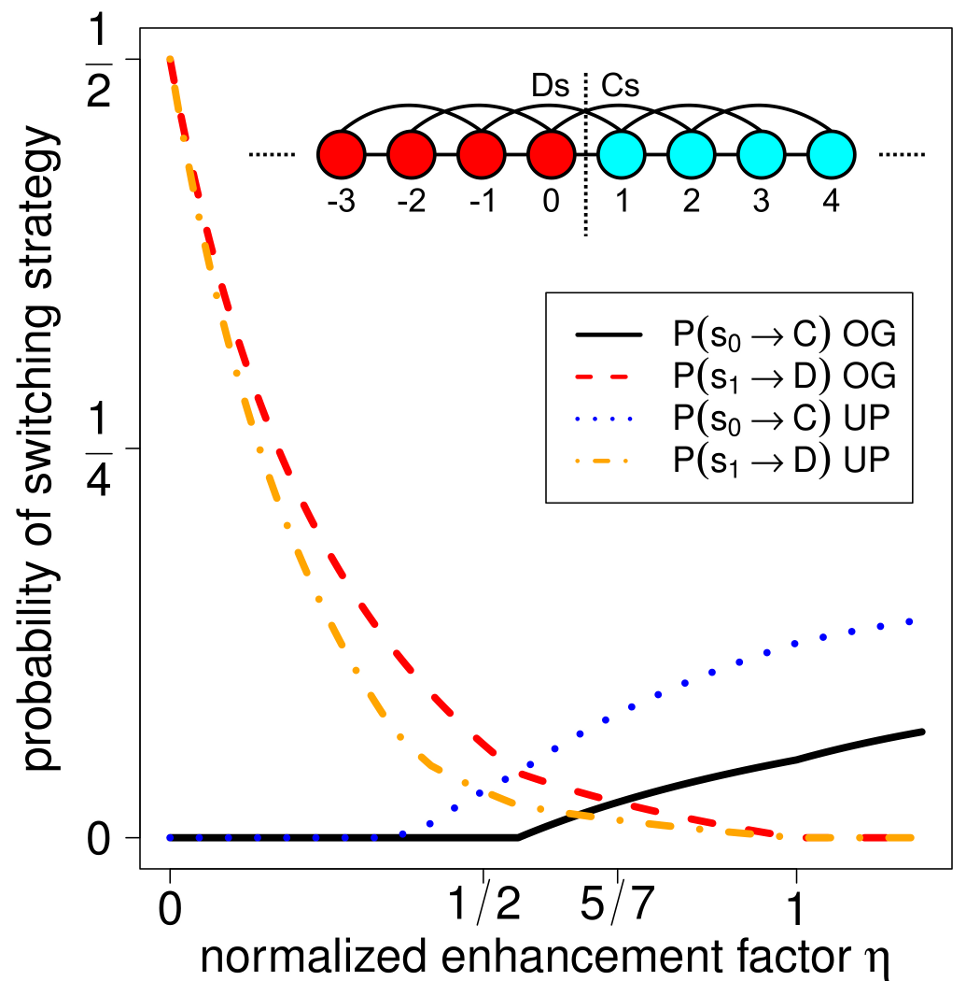

Supplement: Figure S1 — Evolutionary dynamics on rings. In the inset, we plot a ring of degree : the neighborhood of each node comprises the closest two nodes to the left and to the right. Following the graph approach, each node is the center of a game of size five so that each individual ends up interacting with the closest four neighbors to the left and the closest four neighbors to the right. We assume that the initial distribution of strategies is such that nodes are Cs and nodes are Ds. In the main panel, we plot the probabilities of switching strategies for the individuals at the boundary (nodes 0 and 1) when the replacement graph is given by the original graph (OG) and when it is given by the unweighted projection (UP) of the interaction bigraph. As shown, for the graph approach, while for the bigraph approach. See section 1 of Text S1 for the calculation of these probabilities. (TIFF) [file pone.0044514.s001.tiff]

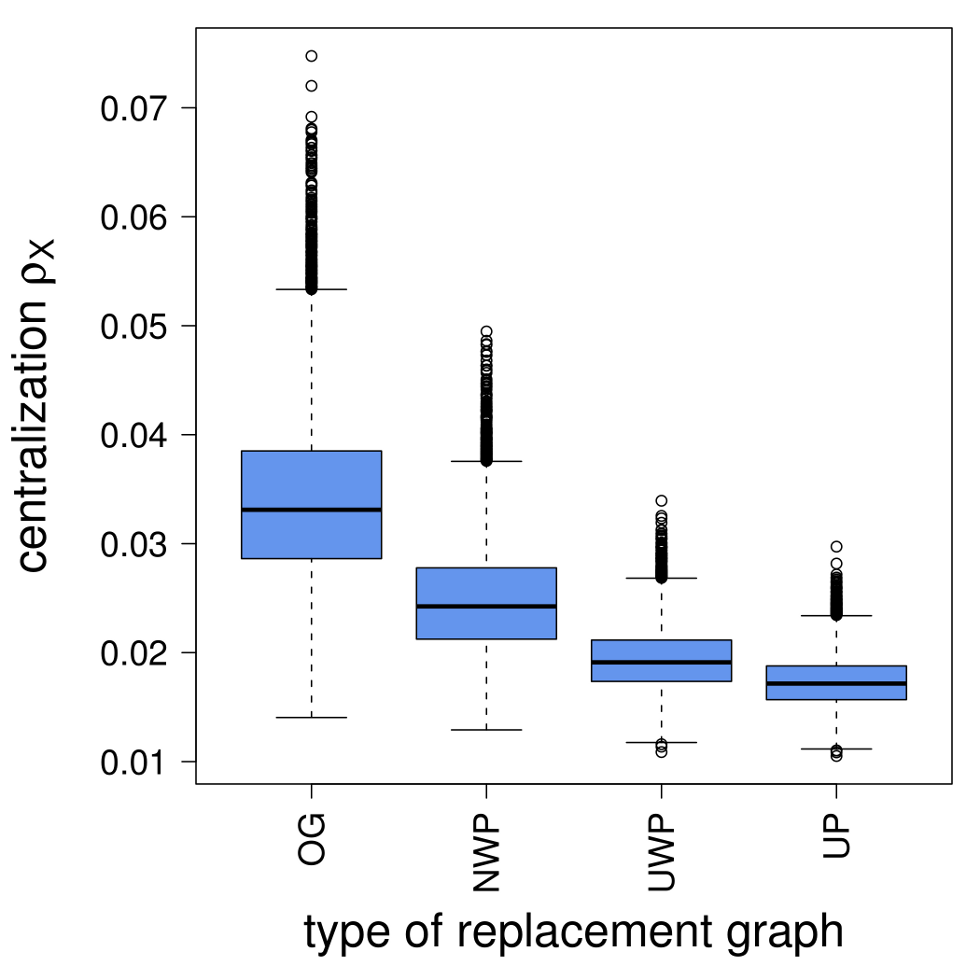

Supplement: Figure S2 — Centralization of the replacement graphs for interaction bigraphs built from Barabási-Albert scale-free networks. Each boxplot shows the distribution of the centralization for a random sample of replacement graphs given by the original graph (OG), the normalized weighted projection (NWP), the unnormalized weighted projection (UWP) and the unweighted projection (UP). In all cases, the original graph is a Barabási-Albert scale-free network of order and mean degree . The projections are taken from bipartite graphs constructed from the original graph using the graph approach. Notice that more centralized networks lead to higher cooperation levels in Panels B and C of Figure 2 in the main text. See section 4 of Text S1 for the definition of the centralization indices used in this figure. (TIFF) [file pone.0044514.s002.tiff]

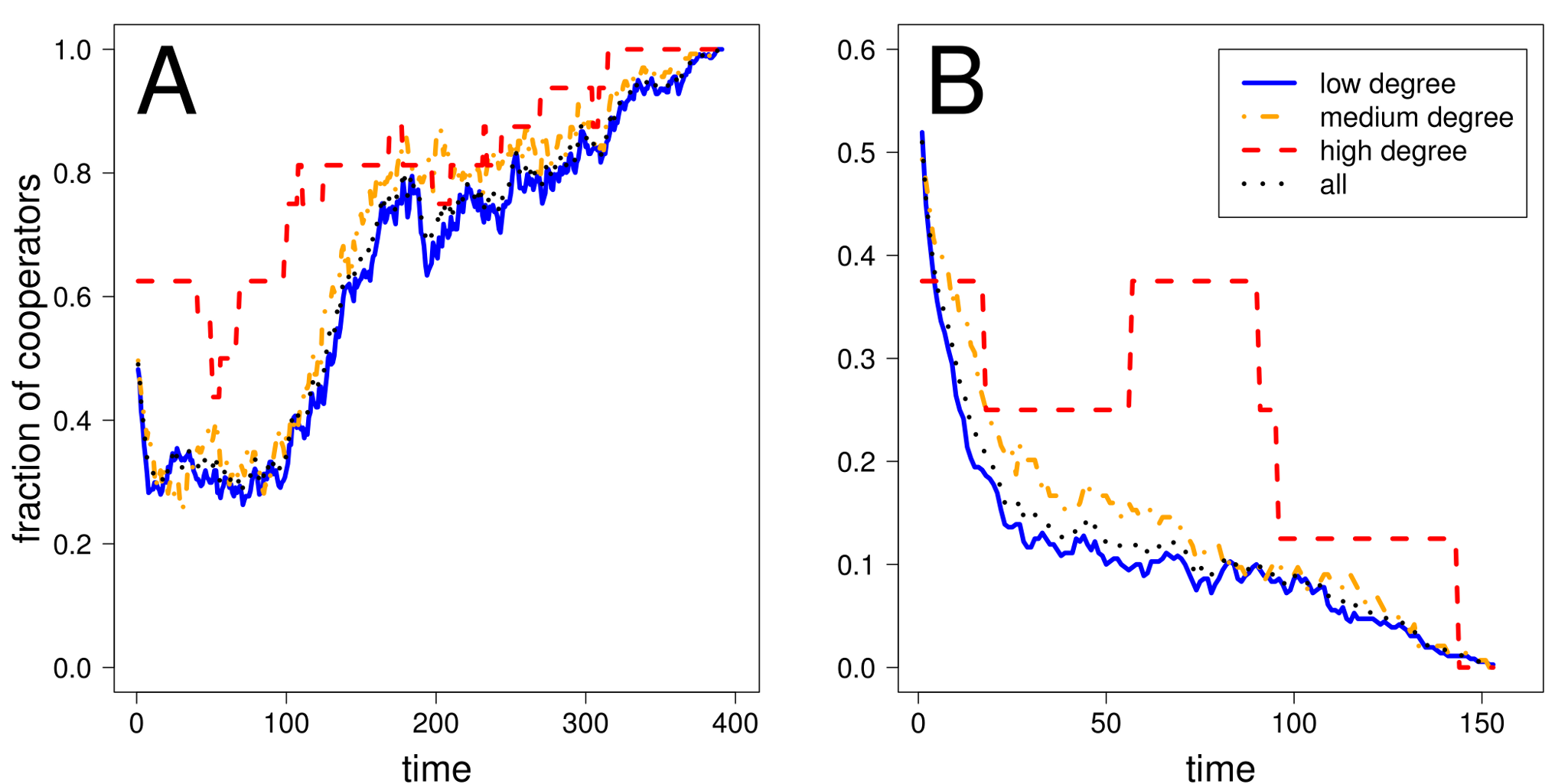

Supplement: Figure S3 — Time-dependence of the fraction of cooperators for different connectivity classes in the config-ba-reg network. The figure shows the fraction of Cs among low-degree (), medium-degree () and high-degree () individuals, for two different simulation runs. In Panel A, initially more than the 60% of the highly-connected individuals are Cs. C-hubs lead the evolutionary process and diffuse cooperative behavior among their less connected neighbors. In Panel B, initially less than 40% of the hubs are Cs. Less connected individuals quickly turn to defection, with medium-degree and high-degree individuals eventually following the trend. Parameters: , and . (TIFF) [file pone.0044514.s003.tiff]

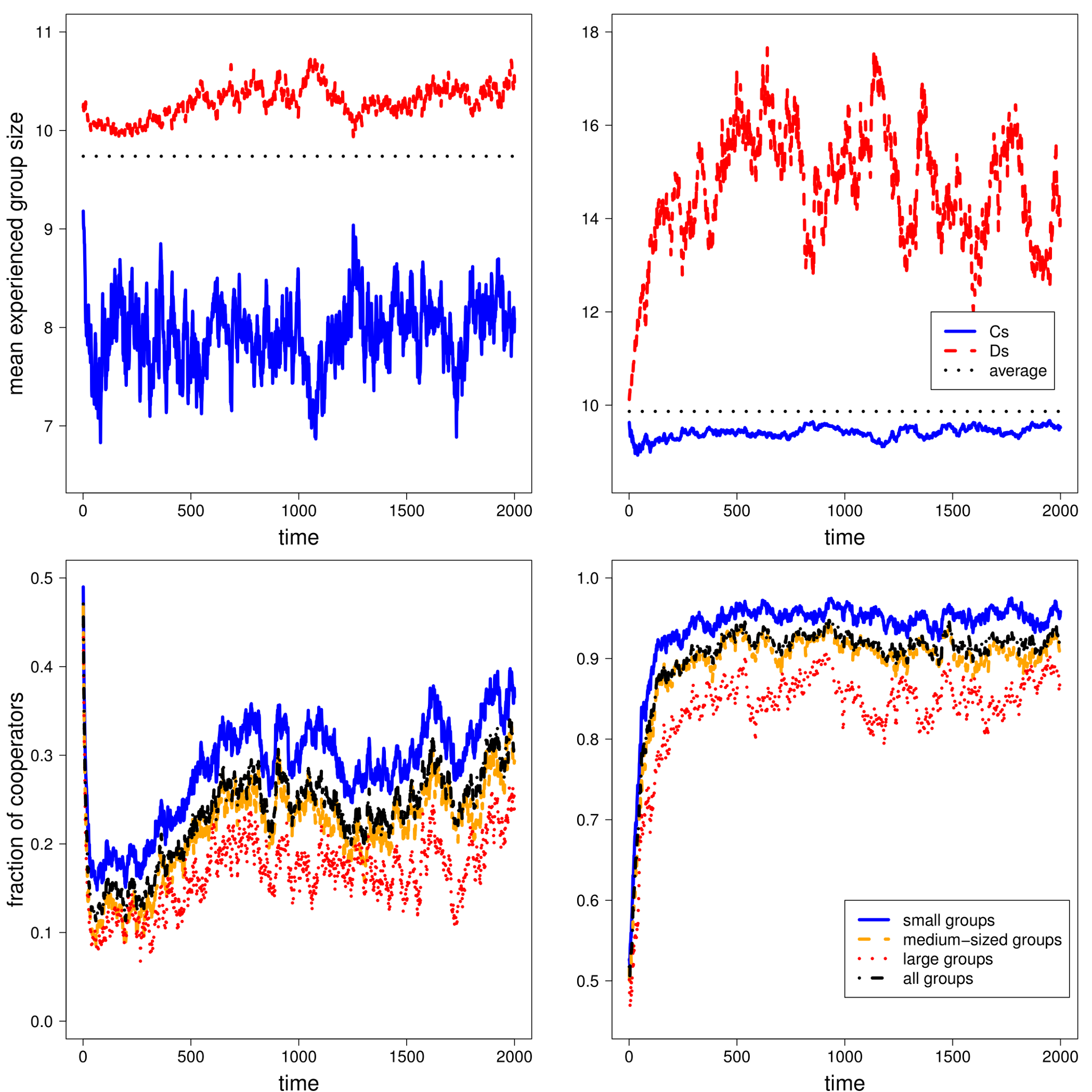

Supplement: Figure S4 — Time-dependence of the average experienced group size and of the fraction of cooperators in groups of different size for config-reg-ba. The figure shows the mean experienced group size for Cs and Ds (top panels) and the fraction of Cs in small (), medium-sized () and large () groups (bottom panels) for (left panels) and (right panels). The evolutionary dynamics on this population structure is such that Cs preferentially cluster together in small groups and Ds cluster together in large groups. Parameters: and . (TIFF) [file pone.0044514.s004.tiff]

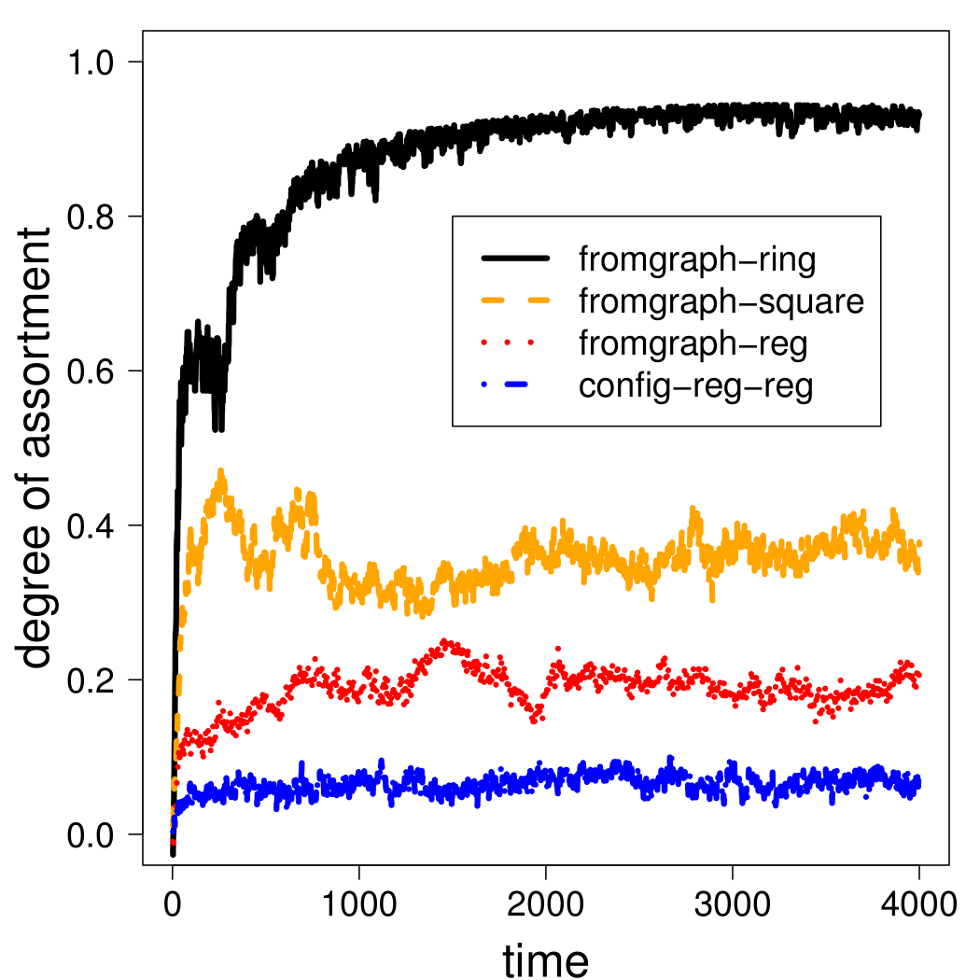

Supplement: Figure S5 — Time evolution of the degree of assortment in the replacement graphs of interaction bigraphs with different bipartite clustering coefficients. The figure shows the time evolution of the degree of assortment in the replacement graph. See section 7 of Text S1 for the definition of degree of assortment we used in this figure. (TIFF) [file pone.0044514.s005.tiff]
