# Supplementary material for: Bipartite Graphs as Models of Population Structures in Evolutionary Multiplayer Games
Source: PLoS One. 2012 Sep 10;7(9):e44514. doi: 10.1371/journal.pone.0044514 (PMC3438187; doi:10.1371/journal.pone.0044514)
Supplement: Text S1 — Supporting Text on analysis of the evolutionary dynamics on rings, stars and double-star graphs, and on definitions of replacement centrality, centralization, bipartite clustering coefficient and degree of assortment. (PDF) [file pone.0044514.s006.pdf]

Bipartite graphs as models of population structures in evolutionary multiplayer games: supporting information text

Jorge Peña*,# and Yannick Rochat†

Institute of Applied Mathematics, Faculty of Social and Political Sciences, University of Lausanne, Lausanne, Switzerland

∗ E-mail: jorge.pena@unibas.ch

# Current address: Faculty of Business and Economics, University of Basel, Basel, Switzerland

† Current address: Institute of Psychology, Faculty of Social and Political Sciences, University of Lausanne, Lausanne, Switzerland

Contents

1 Promotion of cooperation in the ring due to extended replacement neighborhoods 1

2 Evolutionary dynamics on the star graph 3

3 Evolutionary dynamics on the double-star graph 5

4 Replacement centrality 6

5 Replacement centrality on the star graph 7

6 Bipartite clustering coefficient 7

7 Degree of assortment 8

1 Promotion of cooperation in the ring due to extended replacement neighborhoods

In order to understand the origin of the promotion of cooperation in rings shown in Panel A of Figure 2, consider the case of two contiguous clusters of Ds and Cs in a ring of degree $z = 4$. As shown in Figure S1, the probability that the D-player at the C-D boundary (player 0) becomes C is always higher with the larger replacement neighborhood due to the bigraph approach than with the smaller

replacement neighborhood due to the graph approach. This is because in the network given by the graph approach player 0 compares its success with C-players close to the boundary (players 1 and 2), but in the network given by the bigraph approach player 0 can also compare its success with C-players away from the boundary (players 3 and 4), who interact with more Cs than players 1 and 2 and thus obtain larger payoffs. Likewise, the probability that the C-player at the boundary (player 1) changes its strategy to D is always smaller with the larger replacement neighborhood. Defining η_c as the critical value above which the probability that player 0 becomes C is higher than the probability that player 1 becomes D, it is clear that η_c is lower for the bigraph approach ($\eta_c = 1/2$) than for the graph approach ($\eta_c = 5/7 \approx 0.71$). Notice also that, at least for latter case, η_c is a good approximation of the critical value of η for which the cooperation level becomes larger than zero in Panel A of Figure 2.

The calculations of the probabilities of strategy switching leading to Figure S1 are detailed in the following.

Let us denote by $s_i = \{0, 1\}$ the strategy of individual i, with $s_i = 0$ if i is a D and $s_i = 1$ if i is a C. Consider the distribution of strategies in the population shown in the inset of Figure S1. We are interested in the probabilities of strategy switching after one time step for players 0 and 1, i.e. $\Pr(s_i \to 1 - s_i)$ for $i = 0$ and $i = 1$. From the definition of the evolutionary dynamics used in this study, it can be easily shown that, in the case of unweighted replacement graphs, such probability is given by

$$\Pr(s_i \to 1 - s_i) = \frac{1}{|N_H(i)|M} \sum_{j \in N_H(i)} |s_j - s_i| \, (P_j - P_i)\theta(P_j - P_i), \tag{1}$$

where $N_H(i)$ is the open neighborhood of i in the replacement graph H, $|x|$ is the cardinality of x if x is a set or the absolute value of x if x is a number, $\theta(x)$ is the Heaviside step function, such that $\theta(x < 0) = 0$ and $\theta(x \geq 0) = 1$, and $M = P_{max} - P_{min}$, with P_{max} and P_{min} the maximum and minimum possible payoffs. For a ring of degree $z = 4$, and whenever $r < 5$, the maximum possible payoff for a player is that of a D surrounded by Cs and the minimum possible payoff that of a C surrounded by Ds. When $r > 5$ the maximum possible payoff is that of a C surrounded by Cs and the minimum possible payoff that of a D surrounded by Ds. This leads to

$$M = \begin{cases} c(3r + 5) & \text{if } r < 5 \\ 5c(r - 1) & \text{if } r \geq 5 \end{cases} . \tag{2}$$

By calculating the payoffs P_i for $i \in \{-3, -2, \ldots, 3, 4\}$ and using Eq. 1 and Eq. 2 we obtain, in the case the replacement graph is given by the graph approach ($|N_H(i)| = 4\ \forall i$),

$$\Pr(s_0 \to C) = \begin{cases} 0 & \text{if } r < 25/9 \\ \frac{9r-25}{20(3r+5)} & \text{if } 25/9 \leq r < 5 \\ \frac{7r-25}{50(r-1)} & \text{if } r \geq 5 \end{cases}, \tag{3}$$

and

$$\Pr(s_1 \to D) = \begin{cases} \frac{25-7r}{10(3r+5)} & \text{if } r < 25/9 \\ \frac{5-r}{4(3r+5)} & \text{if } 25/9 \leq r < 5 \\ 0 & \text{if } r \geq 5 \end{cases}. \tag{4}$$

Likewise, in the case the replacement graph is the unweighted projection of the interaction bigraph ($|N_H(i)| = 8\ \forall i$):

$$\Pr(s_0 \to C) = \begin{cases} 0 & \text{if } r < 25/14 \\ \frac{14r-25}{40(3r+5)} & \text{if } 25/14 \leq r < 25/12 \\ \frac{13r-25}{20(3r+5)} & \text{if } 25/12 \leq r < 25/9 \\ \frac{7r-15}{8(3r+5)} & \text{if } 25/9 \leq r < 5 \\ \frac{2r-5}{10(r-1)} & \text{if } r \geq 5 \end{cases}, \tag{5}$$

and

$$\Pr(s_1 \to D) = \begin{cases} \frac{5-2r}{2(3r+5)} & \text{if } r < 25/14 \\ \frac{75-26r}{40(3r+5)} & \text{if } 25/14 \leq r < 25/12 \\ \frac{25-7r}{20(3r+5)} & \text{if } 25/12 \leq r < 25/9 \\ \frac{5-r}{8(3r+5)} & \text{if } 25/9 \leq r < 5 \\ 0 & \text{if } r \geq 5 \end{cases}. \tag{6}$$

Equations 3, 4, 5 and 6 are graphically summarized in Figure S1.

2 Evolutionary dynamics on the star graph

Consider a star graph G of order Z comprising one center (h) and $Z-1$ leaves (l) as that shown in Panel A of Figure 3 in the main text. Suppose that the center and m out of the $Z-1$ leaves are Cs. From this graph G, the interaction bigraph B is constructed using the graph approach. The accumulated payoff of

the C-center, of D-leaves and of C-leaves under the distributed NPD are respectively given by

$$\begin{aligned}\Pi(h) &= \frac{rc\left[(m+2)Z^2+2(m-1)Z+4\right]}{4Z^2} - c,\\ \Pi(l^D) &= \frac{rc\left[(m+1)Z+2\right]}{2Z^2},\\ \Pi(l^C) &= \frac{rc\left[Z^2+2Z(m+1)+4\right]}{4Z^2} - c.\end{aligned}$$

The accumulated payoff of the C-center will be higher than that of a D-leaf if

$$\Pi(h) > \Pi(l^D) \Leftrightarrow r > \frac{4}{(m+2)-4/Z},$$

which, for $m = 0$ (the center is the only C) reduces to [1]

$$r > \frac{2}{1-2/Z} \equiv \alpha(Z).$$

Note that α is a monotone decreasing function of Z and that $\alpha = 2$ in the limit of large Z. Consider first the standard case where G (the star graph itself) is taken as the replacement graph H. In this case, and starting with $m = 0$ Cs in the leaves, the C-center invades the whole population for $r > \alpha$, while a D-leaf takes over the C-center if $r < \alpha$ (see Panel C of Figure 3 in the main text). Consider now the case where the replacement graph H is built from the projection of B: H is no longer a star, but a complete graph with different weights attached to the links according to the type of projection used (see Panel D of Figure 3 in the main text). In this case, it can be shown that a D-leaf can invade a C-leaf if $r < 4$. From this, the fate of a single C located on the center depends on r as follows:

1. $r < \alpha$: the C-center is invaded by a defective leaf.

2. $\alpha \leq r < 4$: the C-center invades D-leaves, but C-leaves can be invaded by D-leaves. In any case the system will eventually evolve to a state where all individuals are Cs (this is the only absorbing state), but the time of convergence will depend on the weights of the replacement graph.

3. $r \geq 4$: the C-center and the C-leaves invade D-leaves. The system quickly converges to the all-C state.

3 Evolutionary dynamics on the double-star graph

Following previous related work [1, 2], we consider the evolutionary dynamics on the double-star graph, as it gives hints about the evolutionary dynamics on networks largely dominated by the presence of few hubs, such as scale-free networks. We have two centers, each with $X-2$ and $Y-2$ leaves respectively, plus one edge connecting the two centers. We denote by h_1 and h_2 the two centers and by l_i a leaf connected to center h_i, with $i = 1, 2$ (see Panel A of Figure 4 in the main text). Let us assume that h_1 is a D, h_2 a C, m out of the $X-2$ leaves l_1 are Cs and n out of the $Y-2$ leaves l_2 are Cs. We denote by l_i^C (l_i^D) a leaf l_i with strategy C (D). Their accumulated payoffs under the distributed NPD can be shown to be given by

$$\begin{aligned}
P(h_1) &= \frac{rc(mY+2)}{2XY} + \frac{rc(nY+2)}{2Y^2} + \frac{mrc}{4}, \\
P(l_1^C) &= \frac{rc(mY+2)}{2XY} + \frac{rc}{4} - c, \\
P(l_1^D) &= \frac{rc(mY+2)}{2XY}, \\
P(h_2) &= \frac{rc(mY+2)}{2XY} + \frac{rc(nY+2)}{2Y^2} + \frac{nrc(Y+2)}{4Y} + \frac{rc(Y-2-n)}{2Y} - c, \\
P(l_2^C) &= \frac{rc(nY+2)}{2Y^2} + \frac{rc(Y+2)}{4Y} - c, \\
P(l_2^D) &= \frac{rc(nY+2)}{2Y^2} + \frac{rc}{2Y}.
\end{aligned}$$

Let us consider the case where $m = X-2$ and $n = Y-2$, so that all the individuals in the population are Cs except for the center h_2. If the replacement graph is given by the double star itself (graph approach) leaves compete only with their respective centers, and each center with its leaves and the other center. In this case, it has been shown [1] that the time evolution of the system is such that, typically, the D-strategy spreads preferentially to the leaves of the second star, thus contributing to reducing the fitness of the D-center and to facilitating an invasion from the C-center, after which cooperation easily spreads to the remaining leaves. When the replacement graph is given by the bottom projection of the interaction bigraph (bigraph approach), leaves compete also with other leaves of the same star and with the center of the other star. In this case, the D-center easily invades not only its own leaves but also the leaves of the second star. If $r < 4$, inter-leave competition or social learning eventually leads to the majority of leaves to be Ds and to the D-center to invade the C-center.

4 Replacement centrality

Centrality is a key concept that has been studied in social network analysis since its origins [3], and has since been adopted in other network-related disciplines [4]. Measures of centrality, such as degree, closeness, betweenness and eigenvector centrality, describe individuals' positions in a network relative to others and provide an efficient way of identifying influential actors [5–7]. Likewise, measures of the *centralization* of a network index the tendency of a single node to be more central than other nodes in the network [5].

Here, we generalize the definition of the power index recently proposed in Ref. [8] to weighted networks and define the replacement centrality of node i as

$$\rho_i = \sum_{j \in N_H(i)} \frac{w_{ij}}{\sum_{k \in N_H(j)} w_{jk}}, \tag{7}$$

where $N_H(i)$ is the open neighborhood of node i in the replacement graph and w_{ij} denotes the weight of the link between i and j. According to the evolutionary dynamics considered in this study, ρ_i is equal to the expected number of times individual i is chosen for competition/imitation by other individuals per generation. Other things being equal, replacement centrality measures the importance of an individual in the evolutionary process.

In order to measure the degree of centralization of the network, we make use of the measure used in Ref. [5]:

$$\rho_X = \frac{\sum_{i=1}^{Z} [\rho^* - \rho_i]}{\max \sum_{i=1}^{Z} [\rho^* - \rho_i]},$$

where ρ^* is the largest value of ρ for any node in the network and the maximum operator in the denominator is taken over all possible realizations of a network with Z nodes, so that ρ_X is normalized between 0 and 1. In our case, such maximum is obtained for an unweighted star graph of size Z so that we can write

$$\rho_X = \frac{\sum_{i=1}^{Z} [\rho^* - \rho_i]}{Z(Z-2)}. \tag{8}$$

This is the measure of centralization used for deriving the results shown in Panel F of Figure 3 in the main text and in Figure S2.

5 Replacement centrality on the star graph

Consider a star graph G of order Z with 1 center and $Z-1$ leaves, and the bigraph B built from G via the graph approach. Let the replacement graph H be equal to G (graph approach) or to the projection of the bigraph B (bigraph approach). Then, using Eq. 7 the replacement centrality ρ_h of the center node and the replacement centrality ρ_l of a leaf are respectively given by

$$\rho_h = \frac{(Z-1)w_{hl}}{w_{hl} + (Z-2)w_{ll}}, \tag{9}$$

and

$$\rho_l = \frac{1}{Z-1} + \frac{(Z-2)w_{ll}}{w_{hl} + (Z-2)w_{ll}}, \tag{10}$$

where w_{hl} is the weight of the edge between the center and a leaf and w_{ll} is the weight of the edge between any two leaves. Using Eq. 9, 10 and the values of w_{hl} and w_{ll} given in Panel F of Figure 3 in the main text, we derive the centralization indices shown in Panel F of Figure 3 in the main text.

6 Bipartite clustering coefficient

The bipartite clustering coefficient captures correlations between neighborhoods in a bigraph. In this study, we make use of the definition of bipartite clustering coefficient proposed in Ref. [9]. In order to calculate the bipartite clustering coefficient of the bottom vertices of a bipartite graph, the bipartite clustering coefficient $cc_\bullet(u,v)$ for a pair (u,v) of bottom nodes is defined as their Jaccard similarity coefficient [10]

$$cc_\bullet(u,v) = \frac{|N(u) \cap N(v)|}{|N(u) \cup N(v)|},$$

where $N(u)$ denotes the neighborhood of node u. The bipartite clustering coefficient of a single node is then defined as the sum of Jaccard indices implying this node, divided by the number of nodes at distance 2

$$cc_\bullet(u) = \frac{\sum_{v \in N(N(u))} cc_\bullet(u,v)}{|N(N(u)|}.$$

Finally, the bipartite clustering coefficient of the set of bottom nodes is the average of the bottom vertices' bipartite clustering coefficient,

$$cc_\bullet(\perp) = \frac{\sum_{u\in\perp} cc_\bullet(u)}{|\perp|}.$$

7 Degree of assortment

The degree of assortment r can be defined as the difference between the probability that a random neighbor is a C, given that the focal individual is a C, and the probability that the neighbor is a C, given that the focal individual is a D [11]

$$r = \Pr(C|C) - \Pr(C|D),$$

which can be calculated each time step as

$$r = \frac{2n_{CC}}{2n_{CC} + n_{CD}} - \frac{n_{CD}}{2n_{DD} + n_{CD}},$$

where n_{xy} denotes the number of edges whose extreme points' strategies are x and y.

References

1. Santos FC, Santos MD, Pacheco JM (2008) Social diversity promotes the emergence of cooperation in public goods games. Nature 454: 213–216.

2. Pacheco JM, Pinheiro FL, Santos FC (2009) Population structure induces a symmetry breaking favoring the emergence of cooperation. PLoS Computational Biology 5: e1000596.

3. Bavelas A (1950) Communication patterns in task-oriented groups. Journal of the Acoustical Society of America 22: 725–730.

4. Newman MEJ (2010) Networks: An Introduction. Oxford University Press.

5. Freeman LC (1978) Centrality in social networks conceptual clarification. Social Networks 1: 215–239.

6. Bonacich P (1987) Power and centrality: A family of measures. American Journal of Sociology 92: 1170–1182.

7. Wasserman S, Faust K (1994) Social Network Analysis. Cambridge, UK: Cambridge University Press.

8. Neal J, Neal Z (2011) Power as a structural phenomenon. American Journal of Community Psychology 48: 157–167.

9. Latapy M, Magnien C, Vecchio ND (2008) Basic notions for the analysis of large two-mode networks. Social Networks 30: 31–48.

10. Jaccard P (1901) Étude comparative de la distribution florale dans une portion des Alpes et des Jura. Bulletin de la Société Vaudoise des Sciences Naturelles 37: 547–579.

11. van Veelen M (2009) Group selection, kin selection, altruism and cooperation: When inclusive fitness is right and when it can be wrong. Journal of Theoretical Biology 259: 589–600.
